# Treatment de-escalation for HPV-associated oropharyngeal squamous cell carcinoma with radiotherapy vs. trans-oral surgery (ORATOR2): study protocol for a randomized phase II trial

**DOI:** 10.1186/s12885-020-6607-z

**Published:** 2020-02-14

**Authors:** Anthony C. Nichols, Pencilla Lang, Eitan Prisman, Eric Berthelet, Eric Tran, Sarah Hamilton, Jonn Wu, Kevin Fung, John R. de Almeida, Andrew Bayley, David P. Goldstein, Antoine Eskander, Zain Husain, Houda Bahig, Apostolos Christopoulous, Michael Hier, Khalil Sultanem, Keith Richardson, Alex Mlynarek, Suren Krishnan, Hien Le, John Yoo, S. Danielle MacNeil, Adrian Mendez, Eric Winquist, Nancy Read, Varagur Venkatesan, Sara Kuruvilla, Andrew Warner, Sylvia Mitchell, Martin Corsten, Murali Rajaraman, Stephanie Johnson-Obaseki, Libni Eapen, Michael Odell, Shamir Chandarana, Robyn Banerjee, Joseph Dort, T. Wayne Matthews, Robert Hart, Paul Kerr, Samuel Dowthwaite, Michael Gupta, Han Zhang, Jim Wright, Christina Parker, Bret Wehrli, Keith Kwan, Julie Theurer, David A. Palma

**Affiliations:** 1 Department of Otolaryngology – Head and Neck Surgery, Western University, London, ON Canada; 2 Department of Radiation Oncology, London Health Sciences Centre, Western University, 800 Commissioners Rd. E, London, Ontario N6A 5W9 Canada; 3 Division of Otolaryngology – Head and Neck Surgery, University of British Columbia, Vancouver, BC Canada; 4 Department of Radiation Oncology, University of British Columbia, Vancouver, BC Canada; 5 Department of Otolaryngology – Head and Neck Surgery, University Health Network, University of Toronto, Toronto, ON Canada; 6 Department of Radiation Oncology, University Health Network, University of Toronto, Toronto, ON Canada; 7 Department of Otolaryngology – Head and Neck Surgery, Sunnybrook Health Sciences Centre, University of Toronto, Toronto, ON Canada; 8 Department of Radiation Oncology, Sunnybrook Health Sciences Centre, University of Toronto, Toronto, ON Canada; 9 Department of Radiation Oncology, CHUM, Université de Montréal, Montreal, QC Canada; 10 Department of Otorhinolaryngology - Head and Neck Surgery, CHUM, Université de Montréal, Montreal, QC Canada; 11 Department of Otolaryngology – Head and Neck Surgery, McGill University, Montreal, QC Canada; 12 Department of Radiation Oncology, McGill University, Montreal, QC Canada; 13 Department of Otolaryngology – Head and Neck Surgery, Royal Adelaide Hospital, Adelaide, Australia; 14 Department of Radiation Oncology, Royal Adelaide Hospital, Adelaide, Australia; 15 Department of Medical Oncology, Western University, London, ON Canada; 16 Division of Otolaryngology – Head and Neck Surgery, Dalhousie University, Halifax, NS Canada; 17 Department of Otolaryngology – Head and Neck Surgery, University of Ottawa, Ottawa, ON Canada; 18 Department of Radiation Oncology, University of Ottawa, Ottawa, ON Canada; 19 Section of Otolaryngology – Head and Neck Surgery, University of Calgary, Calgary, AB Canada; 20 Department of Radiation Oncology, University of Calgary, Calgary, AB Canada; 21 Department of Otolaryngology, University of Manitoba, Winnipeg, MB Canada; 22 Department of Otolaryngology – Head and Neck Surgery, Gold Coast University Hospital, Southport, Queensland Australia; 23 Division of Otolaryngology – Head and Neck Surgery, McMaster University, Hamilton, ON Canada; 24 Department of Radiation Oncology, McMaster University, Hamilton, ON Canada; 25 Department of Audiology, London Health Sciences Centre, London, ON Canada; 26 Department of Pathology, Western University, London, ON Canada; 27 School of Communication Sciences and Disorders, Western University, London, ON Canada

**Keywords:** Head and neck cancer, Oropharynx, Transoral surgery, Radiotherapy, Human papillomavirus, Survival, Quality of Life, Randomized controlled trial, De-escalation

## Abstract

**Background:**

Patients with human papillomavirus-positive (HPV+) oropharyngeal squamous cell carcinoma (OPC) have substantially better treatment response and overall survival (OS) than patients with HPV-negative disease. Treatment options for HPV+ OPC can involve either a primary radiotherapy (RT) approach (± concomitant chemotherapy) or a primary surgical approach (± adjuvant radiation) with transoral surgery (TOS). These two treatment paradigms have different spectrums of toxicity. The goals of this study are to assess the OS of two de-escalation approaches (primary radiotherapy and primary TOS) compared to historical control, and to compare survival, toxicity and quality of life (QOL) profiles between the two approaches.

**Methods:**

This is a multicenter phase II study randomizing one hundred and forty patients with T1–2 N0–2 HPV+ OPC in a 1:1 ratio between de-escalated primary radiotherapy (60 Gy) ± concomitant chemotherapy and TOS ± de-escalated adjuvant radiotherapy (50–60 Gy based on risk factors). Patients will be stratified based on smoking status (< 10 vs. ≥ 10 pack-years). The primary endpoint is OS of each arm compared to historical control; we hypothesize that a 2-year OS of 85% or greater will be achieved. Secondary endpoints include progression free survival, QOL and toxicity.

**Discussion:**

This study will provide an assessment of two de-escalation approaches to the treatment of HPV+ OPC on oncologic outcomes, QOL and toxicity. Results will inform the design of future definitive phase III trials.

**Trial Registration:**

Clinicaltrials.gov identifier: NCT03210103. Date of registration: July 6, 2017, Current version: 1.3 on March 15, 2019.

## Background

Oropharyngeal squamous cell carcinoma (OPC) is rapidly increasing in incidence, associated with rising rates of human papillomavirus (HPV) infection [1, 2]. Patients with HPV-positive (HPV+) OPC have substantially better treatment response and overall survival (OS) than patients with HPV-negative (HPV-) disease [3]. Historically the management of OPC has relied on radiotherapy (RT) based approaches as older surgical techniques required large incisions and mandibulotomies with high surgical morbidity and mortality [4]. The addition of concurrent chemotherapy to radiation improved oncological outcomes [5]. However, acute and long term side effects of RT or chemoradiation (CRT) can be severe, including frequent swallowing dysfunction, mucositis, xerostomia, fibrosis, osteoradionecrosis, neutropenia, neurotoxicity and hearing loss [6].

The introduction of minimally invasive transoral surgical techniques including transoral robotic surgery (TORS) and transoral laser microsurgery (TLM) has led to a revival of surgery as the primary treatment of OPC [7, 8]. While primary surgery with transoral surgery (TOS) can avoid some of the side effects of radiotherapy, it can have rare serious consequences such as fatal hemorrhage, stroke, shoulder dysfunction and dysphagia [9].

Currently, there is no level I evidence to favor one treatment strategy over the other. Instead, treatment selection is largely driven by institutional and patient biases with the majority of patients in the United States receiving surgery (82% of T1-T2 disease) [7], while most patients receive primary RT in Canada and Europe [10, 11].

The ORATOR trial is the only trial to examine the question of a primary RT vs. primary TOS approach in a randomized fashion [12]. This phase II trial included 68 patients with OPC regardless of HPV status, and randomized patients to RT (70 Gy, with chemotherapy if N1–2) or TOS plus neck dissection (with or without adjuvant RT/CRT, based on pathology). The primary endpoint was swallowing-related quality of life (QOL) at 1 year, measured using the MD Anderson Dysphagia Inventory (MDADI) [13]. The study found that at 1 year there was a statistically significant difference in swallowing QOL but that this difference did not represent a clinically meaningful change (less than a 10-point difference).

Since the ORATOR trial opened in 2012, the landscape has further shifted to recognize the clinical importance of HPV status. The impact of HPV on outcomes has been so substantial that a separate staging system has been created to better represent the prognosis of these patients [3, 14]. Although not yet part of routine clinical care, research is now focused on de-intensification of treatment in HPV+ OPC, in an attempt to reduce adverse events while maintaining excellent oncologic outcomes. Patients with HPV-related OPC have an excellent chance of survival, and therefore may have to deal with the sequelae of therapy for many decades [3]. With excellent rates of cure, post-treatment QOL becomes of paramount importance. Trials focusing on both primary RT and primary surgical approaches have de-escalated radiotherapy doses; two key trials that are currently underway include NRG-HN-002 and ECOG-3311 [15, 16].

NRG-HN-002 [15] included 308 patients in a phase II parallel-arm design investigating de-intensification in a low-risk p16+ population (T1–2 N1-N2b or T3 N0-N2b as per AJCC 7th edition, and ≤ 10 pack-year smoking history). Arm 1 was conventionally fractionated CRT with 60 Gy (2 Gy/fraction) given in 6 weeks with weekly cisplatin 40 mg/m^2^ and Arm 2 was accelerated RT alone with 60 Gy (2 Gy/fraction) given in 5 weeks with 6 fractions per week. The trial had co-primary endpoints with progression-free survival (PFS) and QOL at 2 years with acceptability criterion of PFS of ≥85% and an MDADI score ≥ 60. Early results presented in abstract format showed the CRT arm met the acceptability criteria for both PFS and MDADI, while the accelerated radiotherapy arm did not meet the PFS acceptability criterion [15].

ECOG-3311 focuses on a primary surgical approach for cT1–2 N1–2b (as per AJCC 7th edition) HPV+ OPC patients, de-escalating the adjuvant RT dose for intermediate risk patients based on surgical pathology. After resection, if a patient has any of: close margins, < 1 mm of extranodal extension (ENE), 2–4 lymph nodes (LN) involved, perineural invasion (PNI) or lymphovascular invasion (LVI) they are randomized in a 1:1 ratio to standard dose adjuvant RT (60 Gy) or de-escalated adjuvant RT (50 Gy). The primary endpoint for this trial is PFS. This trial has completed accrual, but results are not yet reported [16].

Given the dramatic rise in the incidence of HPV disease and the paucity of high-quality data comparing treatment options, the management of HPV+ OPC is arguably the most contentious issue in head and neck oncology [17, 18]. The purpose of this randomized trial is to assess the safety of two de-escalation approaches (primary RT and primary surgery) in early T stage HPV+ OPC by comparing to historical control, and to compare survival, toxicity and QOL profiles between the two approaches. The primary radiotherapy approach is based on the chemoradiation arm of HN002, and the primary surgery approach is based on the treatment paradigm of ECOG-3311 [15, 16].

## Methods/design

The objectives of this trial are to:

1. Compare OS relative to historical controls for de-intensified primary radiotherapy [60 Gy ± chemotherapy] versus TOS and neck dissection [± adjuvant 50 Gy radiotherapy] in patients with early T-stage HPV+ squamous cell carcinoma of the oropharynx.
2. Compare PFS, toxicity and QOL profiles.

Our hypothesis is that for patients with HPV+ T1–2N0–2 (as per AJCC 8th edition) OPC, de-intensified primary RT and primary surgery with de-intensified adjuvant therapy will achieve 2-year OS rates of 85% or greater.

### Study design

**Fig. 1:**
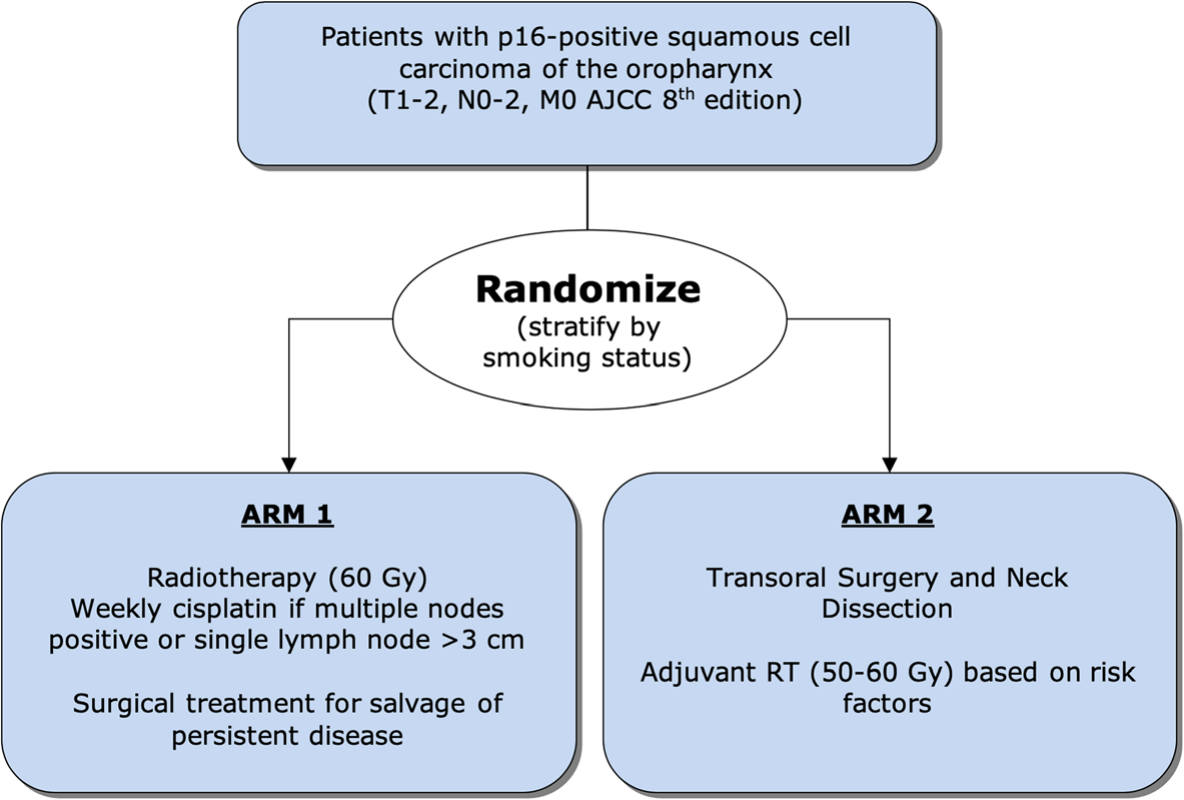
Study Schema

This study is an open-label phase II multi-centre randomized trial, and is designed to assess two potential treatment de-escalation approaches, comparing each to a historical control, with the potential goal of evaluating one or both compared to standard CRT in a subsequent phase III trial. The required sample size is 140 patients. Participating centres will be tertiary, academic hospitals or radiotherapy treatment centres in Canada (updated list of participating centres available on clinicaltrials.gov: identifier NCT03210103). Patients will be randomized between a primary RT-based approach (Arm 1) vs. a primary surgical approach (Arm 2) in a 1:1 ratio using a permutated block design (Fig. 1). There will be one stratification factor: smoking status (< 10 vs. ≥ 10 pack-years). Arm 1 of this trial is based on the chemoradiation arm of HN002 (60 Gy ± concomitant weekly cisplatin based on clinical nodal disease) [15], and Arm 2 is similar to the treatment paradigm of ECOG-3311 (TOS ± adjuvant RT (50–60 Gy) based on risk factors) [16].

### Primary endpoint

- OS
  - ◦ Defined as time from randomization to death from any cause

This study was originally launched with a primary endpoint of PFS. The results of the original ORATOR trial became available in February 2019. These suggested that OS would be a preferred endpoint for ORATOR2 as both arms in ORATOR showed excellent OS in p16+ cancers (both > 92% at 2 years). OS is preferred as the primary endpoint to evaluate de-escalation, since it was evident in ORATOR that progression events, whether local, regional, or distant, can often be salvaged for cure with surgery, radiation, or systemic therapy including immunotherapy. Therefore, in Feb 2019, without knowledge of outcomes data from ORATOR2, this trial was amended to promote OS from a secondary to primary endpoint, and demote PFS to a secondary endpoint.

### Secondary endpoints

- 2-year PFS (comparison with historical controls)
  - ◦ Time from randomization to disease progression at any site or death PFS events are defined as death from any cause, or first recurrence of tumor at any site (including local, regional, or distant). Second primary tumors (e.g. head and neck cancer at a different site, such as laryngeal cancer) will not be included as PFS events.
- 2-year OS and PFS comparisons between Arm 1 and Arm 2
- Swallowing-related QOL at 1-year post-treatment
  - ◦ Assessed using the MDADI
- QOL at other time points
  - ◦ Assessed using the MDADI, the European Organisation for Research and Treatment of Cancer (EORTC) Quality of Life Cancer Patients general (QLQ-C30) and head & neck (H&N35) scales, the Voice Handicap Index (VHI-10), the Neck Dissection Impairment Index (NDII), the Patient Neurotoxicity Questionnaire (PNQ), and the EuroQOL 5-Dimension 5-Level (EQ-5D-5 L).
- Toxicity
  - ◦ Assessed by the National Cancer Institute Common Toxicity Criteria (NCI-CTC) version 4
- Other functional measurements, including, measured by:
  - ◦ Feeding tube rate at 1-year
  - ◦ Common Toxicity Criteria for Adverse Events (CTC-AE) Dysphagia scores

### Inclusion criteria

- Minimum age 18
- Willing to provide informed consent
- Eastern Cooperative Oncology Group (ECOG) performance status 0–2
- Histologically confirmed squamous cell carcinoma
- HPV+ tumor, as determined by: positive p16 status, real-time polymerase chain reaction (PCR) or in-situ hybridization. Central confirmation is not required.
- Primary tumor site in the oropharynx (includes tonsil, base of tongue, soft palate, walls of oropharynx)
- Eligible for curative intent treatment, with likely negative resection margins at surgery. For patients where adequate transoral access is in question, they will first have an examination under anesthesia ensure adequate exposure can be obtained prior to randomization.
- Smokers and non-smokers are included. Patients will be stratified by < 10 versus ≥10 pack-year smoking history. Pack-years are calculated by multiplying the number of years smoked by the number of packs of cigarettes smoked per day. One pack is considered to contain 20 cigarettes.
- Tumor stage (AJCC 8th edition): T1 or T2
- Nodal stage (AJCC 8th edition): N0, N1 or N2
- For patients who may require chemotherapy (i.e. patients with multiple lymph nodes positive or a single node more than 3 cm in size, in any plane; see section 6): complete blood count/differential obtained within 4 weeks prior to randomization, with adequate bone marrow function, hepatic, and renal function, defined as: Absolute neutrophil count > 1.5 × 10^9^/L; Hemoglobin > 80 g/L; platelets > 100 × 10^9^/L; Bilirubin < 35 μmol/L; AST, ALT < 3 x the upper limit of normal; serum creatinine < 130 μmol/L or creatinine clearance ≥50 mL/min
- Head and neck multidisciplinary clinic (radiation oncologist and surgeon) and multidisciplinary tumor board presentation prior to randomization.

### Exclusion criteria

- Unambiguous clinical or radiological evidence of ENE on pre-treatment imaging. This includes the presence of matted nodes, defined as 3 or more nodes that are abutting with loss of intervening fat planes.
- Serious medical comorbidities or other contraindications to RT, surgery or chemotherapy
- Inability to attend full course of RT or follow-up visits
- History of previous head and neck RT or previous head and neck cancer within 5 years
- Metastatic disease present
- Prior invasive malignant disease within 5 years, with the exception of non-melanoma skin cancer
- Lactating or pregnant women

### Pre-treatment evaluation

The following evaluations are required:

- History and physical examination (including laryngopharyngoscopy) by a radiation oncologist and head and neck surgeon within 8 weeks prior to randomization
- For patients where adequate transoral access is in question, they will have an examination under anesthesia to ensure adequate exposure can be obtained prior to randomization
- Staging imaging within 12 weeks prior to randomization: Contrast-enhanced CT of the neck and chest or MRI of the neck with CT of the chest or whole body PET/CT
- Documentation of smoking history
- Histological confirmation of squamous cell carcinoma
- p16+ or HPV+ tumor status, as defined above
- Dental evaluation within 6 weeks prior to randomization
- Audiogram before initiation of treatment, with baseline CTCAE grade assessment
- Assessment of all baseline symptoms, including assessment of dysphagia, using CTC-AE version 4 within 2 weeks prior to randomization. Baseline dysphagia CTC-AE will be scored in all patients.
- Completion of QOL scoring within 2 weeks of randomization
- CBC/differential, hepatic (AST, ALT, total bilirubin) and renal function testing (BUN and creatinine, or creatinine clearance) within 4 weeks prior to randomization, if chemotherapy would be required
- Pregnancy test for women of child-bearing age, within 2 weeks prior to randomization
- Blood sample for whole genome sequencing analysis prior to initiation of treatment
- Informed consents must be obtained prior to any study specific activities

### Interventions

Initiation of treatment should occur within 4 weeks of randomization.

#### Primary radiotherapy (Arm 1)

Treatment in this arm is generally based on Arm 1 of NRG-HN002 [15], and may consist of either radiotherapy alone or concurrent chemoradiation depending on the patient’s clinical nodal status (see Table 1).

**Table 1:** Delivery of radiation ± chemotherapy depending on clinical nodal status in Arm 1 (Primary RT)

	Radiation Alone: Accelerated radiation	Concurrent chemotherapy: Weekly cisplatin 40 mg/m^2^ for 6 cycles
Nodal status	Node negative (N0) OR Single node less than 3 cm in maximal diameter	Multiple lymph nodes OR Single lymph node more than 3 cm in maximal diameter
Fractionation	Radiation over 5 weeks, with 6 fractions a week 6th weekly fraction given on a weekday with a minimum 6 h intrafraction interval, or on a Saturday	Daily radiation, Monday-Friday over 6 weeks
Special conditions	In patients > 70 years of age, standard fractionation (daily, Monday-Friday over 6 weeks) can be used at the discretion of the radiation oncologist	In patients who are deemed unfit for weekly cisplatin, the dose and/or schedule can be modified, or cetuximab or weekly carboplatin AUC 1.5 can be used, at the discretion of the medical oncologist.

Dose levels are as follows:

- 60 Gy in 30 fractions: Gross Tumor and Involved Nodes
- 54 Gy in 30 fractions: High risk subclinical areas.
- 48 Gy in 30 fractions: Low-risk nodal areas

Specific radiotherapy volume definitions for Arm 1 are described in Table 2. In all cases a 5 mm CTV to PTV expansion is to be used.

**Table 2:** Specific RT volume definition volumes for Arm 1 (Primary RT)

Radiotherapy Volume	Definition
GTV_P	Gross tumour volume
GTV_N	Gross nodes: – > 1.5 cm long axis – > 1 cm short axis – Necrotic – PET positive
CTV60	Combination of GTV_P and GTV_N with a 5 mm expansion, excluding natural boundaries of spread
CTV54	– A 1 cm expansion on the GTV_P – Any nodal level that contains a positive node. – Any node < 1 cm in short axis the radiation oncologist deems suspicious for harbouring disease. This node plus an additional 5 mm margin will be included in the CTV54. – The first echelon draining nodal levels. This is nearly always level 2, but should include the lateral retropharyngeal nodes (RP) for soft palate and posterior pharyngeal wall extension.
CTV48	– Patients that are node negative: ◦ Ipsilateral: II-IV. RP only if extension to posterior pharyngeal wall or soft palate ◦ Contralateral^a^: II-IV, RP only if extension to posterior pharyngeal wall or soft palate – All patients with N1 (ipsilateral) nodal disease: ◦ Ipsilateral: Ib, II-V, RP ◦ Contralateral^a^: II-IV, RP only if extension to posterior pharyngeal wall or soft palate – All patients with N2 disease: ◦ Ipsilateral and contralateral: Ib, II-V, RP

^a^If treating the contralateral neck

Treatment breaks, early RT termination and discontinuation of systemic therapy is at the discretion of the treating oncologist based on patient treatment toxicity.

##### Salvage surgery

Treatment response will be evaluated 10–12 weeks after completion of RT. This can be done using a CT scan and/or a PET-CT scan.

Treatment of residual disease at the site of the primary tumor will be determined by the treating physicians, and should include surgical salvage if feasible.

Management of residual enlarged lymph nodes in the neck should be guided by standard institutional practice. In general, for patients with residual enlarged nodes on CT, a PET-CT is preferred to confirm fluorodeoxyglucose (FDG) avidity prior to neck dissection. If the PET-CT is negative in the setting of enlarged nodes on CT, then close interval follow-up with repeat CT every 2–3 months is recommended until the lymph nodes resolve. If PET-CT is unavailable, any nodes > 1 cm in short axis should, at a minimum, be carefully followed with repeat CT every 2–3 months until the lymph nodes resolve, with neck dissection at the discretion of the treating physician.

Salvage surgery for the primary tumor or lymph nodes within 5 months of treatment will be considered part of the initial treatment package and scored as persistent disease, not as recurrence. Surgery beyond 5 months post-treatment will be scored as recurrence if malignancy is evident in the pathology specimen.

#### Primary TOS (Arm 2)

Patients with easily accessible oropharyngeal tumors (determined by the consulting surgeon), will proceed directly to TOS. If adequate transoral access is in question, patients will undergo an examination under anesthesia to ensure adequate exposure can be obtained prior to randomization.

Surgical resection will be carried out with at least 1 cm margins. At the time of surgery circumferential margins will be taken and sent for frozen section. The resection will be revised until negative margins are obtained if feasible. Wounds may be closed by primary closure, local flaps (i.e. buccal or palatal flaps) or allowed to heal by secondary intention at the discretion of the treating surgeons. Free flap and regional flaps are not allowed.

Standard selective neck dissections for the lymph node areas at risk will be performed at the time of transoral resection, or as a staged procedure 2 weeks prior to the primary site resection. At this time the lingual and facial branches of the ipsilateral external carotid artery must be ligated on the side ipsilateral to the primary tumor. Patients with tonsillar, lateral palate and lateral pharyngeal cancers, with < 1 cm of base of tongue or palate extension, will undergo ipsilateral neck dissections only. All other patients will undergo bilateral neck dissections. Selective neck dissections will be limited to levels 2–4, unless levels 1 or 5 are involved.

If there is a positive margin at the time of TOS at the primary site, an attempt to clear the positive margin transorally will be allowed. If a positive or close margin is found on the final pathology from the transoral resection, an attempt to clear the margin transorally is allowed within 4 weeks of the original TOS resection. This can be done with or without the robot at the surgeon’s discretion.

A tracheostomy is strongly recommended, but not mandatory, to provide airway protection in the event of swelling and/or bleeding.

##### Adjuvant radiotherapy

Adjuvant radiotherapy will be determined based on pathological findings. No more than 6 weeks should elapse between the date of surgery and the initiation of adjuvant therapy.

Adjuvant radiotherapy is required for the following risk factors:

- ENE
- Positive margins or close resection margins (< 3 mm)
- More than 1 lymph node positive, or any lymph node > 3 cm in size on pathology
- LVI
- pT3–4 disease

In situations where PNI alone is present, without the other risk factors above, adjuvant RT is at the discretion of the treating physicians.

Patients with positive margins or ENE will receive a 6-week course of radiation as follows:

- 60 Gy in 30 fractions: Area of positive margins or ENE
- 54 Gy in 30 fractions: Operative bed, including primary tumor location and all dissected nodal levels
- 48 Gy in 30 fractions: Undissected areas considered to be at low-risk of harbouring microscopic disease.

Patients without positive margins or ENE will receive a 5-week course of radiation as follows:

- 50 Gy in 25 fractions: Operative bed, including primary tumor location and all dissected nodal levels
- 45 Gy in 25 fractions: Undissected areas considered to be at low-risk of harbouring microscopic disease.

Concurrent chemoradiation will not be delivered in the adjuvant setting unless gross tumor is left behind at the primary site or in the neck AND the patient would have received chemotherapy had they been randomized to Arm 1. Chemotherapy is NOT used for patients with ENE or positive margins. Retrospective surgical data do not support an OS benefit to the use of chemotherapy in HPV+ patients with ENE [19].

Specific radiotherapy volume definitions for Arm 2 are described in Table 3. In all cases a 5 mm CTV to PTV expansion is to be used.

**Table 3:** Specific RT volume definition volumes for Arm 2 (Primary TOS) if adjuvant RT is required

Radiotherapy Volume		Definition
With ENE or positive margins (30 fractions over 6 weeks)	Without ENE or positive margins (25 fractions over 5 weeks)	
CTV60		Areas of positive margins and/or ENE
CTV54	CTV50	Entire tumor bed and any dissected neck nodal levels
CTV48	CTV45	Undissected nodal areas that must be treated based on pathological results. Treatment volumes must include nodal levels adjacent to areas containing involved nodes (eg. if there is a level II node positive, levels Ib and V must be included) – All patients with N1 (ipsilateral) nodal disease: ◦Ipsilateral: Ib, II-V, RP ◦Contralateral^a^: II-IV, RP only if extension to posterior pharyngeal wall or soft palate – All patients with N2 disease: ◦Ipsilateral and contralateral: Ib, II-V, RP

^a^If treating the contralateral neck

The AJCC 8th edition has a pathologically based staging system for HPV+ patients treated with primary surgery that differs significantly from the clinically based staging system used for patients treated with primary radiation. All surgically treated patients will be separately re-staged after the final pathology is available.

In the unlikely event of residual gross disease, the patient should then receive adjuvant treatment using the dose fractionations in Arm 1. In the unlikely scenario where a patient is deemed to have highly aggressive disease (e.g. frank growth/progression during the post-surgical interval), the radiation oncologist may elect to treat with a standard (non-de-escalated) dose of 70 Gy in 35 fractions. Treatment breaks, early RT termination and discontinuation of systemic therapy is at the discretion of the treating oncologist based on patient treatment toxicity.

### Unilateral vs. Bilateral radiation

Unilateral radiation is recommended if the following criteria are ALL met:

- tonsil primary
- < 1 cm extension into the tongue base or palate
- no posterior pharyngeal wall extension
- no ENE
- N0, or only a single ipsilateral lymph node positive

Unilateral radiation is optional if the following criteria are ALL met

- tonsil primary
- < 1 cm extension into the tongue base or palate
- no posterior pharyngeal wall extension
- no ENE
- more than one ipsilateral lymph node positive, but are all less than 6 cm, and are all in level II.

In all other cases, bilateral radiation is mandatory. These criteria apply to all patients in Arm 1, and to patients in Arm 2 who require adjuvant RT. For the patients in Arm 2 who receive adjuvant RT, these criteria are based on the pathological findings and intraoperative findings, not the pre-operative clinical findings.

### Radiotherapy technique, immobilization, localization and planning

Intensity modulated radiotherapy (IMRT) will be used for all patients in this study. IMRT can be delivered using static-beam techniques or rotational techniques (e.g. Tomotherapy or Volumetric Modulated Arc Therapy [VMAT]). A custom thermoplastic shell will be used to immobilize all patients. Patients will then undergo a planning CT simulation (head and neck to below the clavicles, slice thickness 3 mm or less.) Patients in Arm 1 will receive contrast (unless contraindicated). For patients in both arms, when necessary, the planning CT will be fused with other diagnostic imaging).

Organ at risk (OAR) contouring definitions, dose constraints and planning priorities are shown in Additional file 1, adapted from RTOG protocols 1016 [20] (Arm 1) and 0920 [21] (Arm 2), HN-002 [15], ECOG-3311 [16] and the NCIC-CTG HN6 protocol [22]. Dose constraints are the same whether 25 or 30 fractions are delivered, as the radiobiological conversion factor is small.

Plans will be normalized to ensure that 95% of each PTV is covered by 100% of the prescription dose for that volume. 99% of each PTV must receive at least 93% of the prescription dose. The maximum dose must be less than 115% of the highest prescription dose.

### Quality assurance

#### Radiotherapy quality assurance

A quality assurance protocol is used to ensure safe and efficacious treatment with the following elements present for each patient:

- Discussion of each radiotherapy plan at head and neck quality assurance (QA) rounds prior to, or within the first week of treatment
- Physics staff will confirm all dose delivery for IMRT plans (including arc-based treatments) before treatment.
- Cone-beam CT and/or orthogonal x-rays will be used daily to verify treatment positioning, as per institutional standard practice.
- Prior to enrolling patients, each centre will be given a sample CT dataset for contouring, planning and physics QA. Enrollment can begin once the plan and QA have been approved at the London Regional Cancer Program (LRCP).

#### Surgery quality assurance

The learning curve for surgeons carrying out transoral oropharyngeal cancer resections has been demonstrated to be short for early-stage cases, with significant improvements in operative time after 20 cases (but not oncologic outcomes) as learning occurs [23]. Surgeons will be required to complete a “Surgical Credentialing Questionnaire” based closely on the ECOG-3311 credentialing criteria (Additional file 2). This includes 1) being fellowship trained in head and neck surgical oncology, 2) having carried out at least 20 transoral oropharyngeal cancer resections as primary surgeon, 3) providing operative notes for 10 of those cases, 4) a minimum of 5 oropharyngeal resections in the last year and 5) perform at least 30 neck dissections per year.

Individual surgeons will be reviewed for surgical quality after every 5 surgical cases by the principal investigator (AN). Bleeding or positive margin rates of greater than 20% may result in exclusion from the trial at the discretion of the principal investigators. The occurrence of an oropharyngeal bleeding fatality or severe anoxic brain injury in the absence of a tracheostomy may also result in the exclusion of the centre from the trial.

Centres will be reviewed for surgical quality after 5, 10 and 15 surgical cases by the principal investigators. Bleeding or positive margin rates of greater than 20% may result in exclusion from the trial at the discretion of the principal investigators. The occurrence of an oropharyngeal bleeding fatality or severe anoxic brain injury in the absence of a tracheostomy may also result in the exclusion of the centre from the trial.

Pathology reporting of ENE: The electronic case report forms must include a description of ENE using the same descripTOS as the ECOG-3311 trial:

- **absent** (node without metastasis or nodal metastasis with smooth/rounded leading edge confined to thickened capsule/pseudocapsule)
- **minimal** (tumor extends ≤1 mm beyond the lymph node capsule)
- **present - extensive** (tumor extends > 1 mm beyond the lymph node capsule (includes soft tissue metastasis))

### Subject discontinuation / withdrawal

Subjects may voluntarily discontinue participation in the study at any time. If a subject is removed from the study, the clinical and laboratory evaluations that would have been performed at the end of the study should be obtained. If a subject is removed because of an adverse event, they should remain under medical observation as long as deemed appropriate by the treating physician.

### Follow-up evaluation

Day 1 of follow-up will be the first day of radiotherapy (Arm 1) or the date of surgery (Arm 2); however, survival will be calculated from the date of randomization. The follow-up schedule is summarized in Additional file 3, and is the same as the follow-up schedule of ORATOR [24]. On Arm 1 patients will be seen during treatment, for a post-treatment clinical assessment at 4–6 weeks. Post-treatment imaging with a CT or PET/CT of the neck will be obtained. On Arm 2 a post-operative assessment will occur at 2 weeks with adjuvant radiotherapy beginning within 6 weeks of surgery if required. If radiotherapy is delivered the same treatment and post-treatment assessments will occur as on Arm 1, A return visit with the surgeon will occur at 3 months from the date of surgery. On both arms patients will be seen every 3 months up to 2 years and then every 6 months thereafter (up to 5 years) from the start of treatment, with clinical, toxicity and QOL assessments (Additional file 3). For patients in both arms, a CT of the neck, and chest, MRI of the neck with CT of the chest or whole body PET/CT will be obtained at 12 months. Additional imaging or laboratory investigations and additional treatment (eg. Salvage treatment) will be carried out at the discretion of the treating physicians.

#### Disease progression and new primary

In the event of disease progression, the details of new or recurrent disease and treatment details will be captured in the case report form. Audiologic assessments, bloodwork and QOL questionnaires should continue to be completed according to the follow up schedule (Additional file 3). All ongoing adverse events (AEs) at the time of progression should be followed until resolution. Subsequent imaging after progression can be completed at the discretion of the treating investigator.

##### Salvage surgery after primary radiotherapy

Treatment of residual disease at the site of the primary tumor will be determined by the treating physicians, and should include surgical salvage if feasible.

Management of residual enlarged lymph nodes in the neck should be guided by standard institutional practice. In general, for patients with residual enlarged nodes on CT, a PET-CT is preferred to confirm FDG avidity prior to neck dissection. If the PET-CT is negative in the setting of enlarged nodes on CT, then close interval follow-up with repeat CT every 2–3 months is recommended until the lymph nodes resolve. If PET-CT is unavailable, any nodes > 1 cm in short axis should, at a minimum, be carefully followed with repeat CT every 2–3 months until the lymph nodes resolve, with neck dissection at the discretion of the treating physician.

Salvage surgery for the primary tumor or lymph nodes within 5 months of treatment will be considered part of the initial treatment package and scored as persistent disease, not as recurrence. Surgery beyond 5 months post-treatment will be scored as recurrence if malignancy is evident in the pathology specimen.

### Measurement of outcomes

- Survival outcomes:
  - ◦ OS: time from randomization until death from any cause
  - ◦ PFS: time from randomization to either progression or death, whichever occurs first.
- QOL outcomes (measured at baseline and at 6-month intervals except PNQ):
  - ◦ MDADI
  - ◦ EORTC QLQ-C30 and H&N35 scales
  - ◦ NDII
  - ◦ VHI-10
  - ◦ PNQ will be completed at 1 year post-treatment.
- Economic assessment:
  - ◦ EQ-5D-5 L: administered at baseline and 6 month intervals. Quality adjusted life years (QALYs) will be assessed as the area under the preference-weighted survival curve. Overall costs of each treatment strategy will be abstracted from the available literature. The incremental cost effectiveness ratios (ICERs) between treatment arms will be compared through the standard method of ratio between differences in costs and QALYs. Point estimates for these differences can be derived from multivariable generalized estimating equations (GEE) or general linear model (GLM) analyses.
- Toxicity outcomes:
  - ◦ CTC-AE toxicities will be recorded during treatment and at every follow-up visit.

### Statistical considerations

#### Randomization

Patients will be randomized between Arm 1: Arm 2 stratified based on smoking status (< 10 pack-years vs. ≥10 years) in a 1:1 ratio in a permutated block design. The randomization sequence is known only to the statistician and uploaded into a restricted-access database (REDCap) housed on secure hospital servers at LHSC [25]. Upon enrollment of a patient, the database will be accessed by the trial coordinator to obtain the next intervention in the random sequence, for the pertinent stratum, which will then be assigned to the patient.

#### Sample size calculation

The 2-year OS in each arm, based on the results of ORATOR, is estimated to be 94%. A 2-year OS of < 85% will be considered inadequate. In order to differentiate an OS of 94% vs. 84% using a 1-sided one-sample binomial test, with 80% power and alpha of 0.05, with 10% dropout, 70 patients are needed in each arm (140 total).

#### Analysis plan

Patients will be analyzed in the groups to which they are assigned (intention-to-treat). Comparisons of OS and PFS with historical controls will be evaluated using a one-sided binomial test. The 2-year PFS in this cohort is estimated to be approximately 85%, based on the results of CCTG HN6 [22]. If the 2-year PFS is < 75%, it will be considered unacceptable. Therefore, PFS in each arm will be compared against a benchmark PFS of 74%. A comparison of OS and PFS between the two treatment arms will also be conducted, using the stratified log-rank test (stratified by smoking pack-year history). With a sample size of 140 patients, we will have 80% power to detect a 10% superiority in OS in either arm (assuming baseline OS of 94% in whichever arm is superior), using a two-sided alpha of 0.05. A two-sample T-test will be used to compare QOL scores at 1-year (excluding PNQ). The percentage of patients in each arm who experience a clinically significant QOL decline (10 points) will also be reported. Pre-planned subgroup analysis will occur based on the stratification variable (smoking pack-year history). A Cox proportional hazards multivariable regression analysis will be used to determine baseline factors predictive of survival. QOL analysis for secondary endpoints will be performed in the same way as the ORATOR trial using linear mixed-effects models [24]. The original ORATOR trial will be used for additional historical controls [12]. A comparison will occur between HPV+ patients in ORATOR (who were treated with more aggressive approaches) and ORATOR2 to assess differences in QOL and time-to-event outcomes.

### Data safety monitoring committee

The data safety monitoring committee (DSMC), consisting of at least one surgical oncologist, one radiation oncologist, and one medical oncologist not involved in the study and without competing interests, will meet bi-annually after study initiation to review toxicity outcomes. The DSMC can recommend modification of the trial based on toxicity outcomes.

After half of the patients are enrolled and followed for 6 months, one interim analysis will take place. For this interim analysis, OS at 2-years will be calculated for each arm. The DSMC may recommend cessation or modification of the trial if any of these two criteria are met:

1. The rate of grade 5 toxicity definitely related to treatment is > 5% in either arm
2. The upper bound of the 95% confidence interval of OS at 2 years does not include 94%.

#### Biomarker studies

All of the biomarker studies described in this section will be performed in Dr. Anthony Nichols’ laboratory located at the London Regional Cancer Program, in London, Ontario. All specimens will be labeled solely with the patient’s unique study identifier number, stored in a secure facility at London Health Sciences Centre (LHSC), and will be accessed only by clinical trial staff. Details are provided in Additional file 4. Ten mL of blood shall be drawn pre-operatively into a heparinized (green top) tube and directly transported to Dr. Nichols’ lab. At the end of the study pre-treatment formalin fixed paraffin embedded (FFPE) primary site biopsy specimens will be retrieved in 10 slides 8 μm thick as well as three 1 mm core punch biopsies from the FFPE blocks and transported to Dr. Nichols’ lab. For patients treated at the lead centre (LRCP) and randomized to primary surgery a fresh frozen specimen will be collected. Specifically, at the time of operation the main specimen will be taken to pathology frozen section room and a portion from the center of the specimen will be taken with the assistance of the pathologist and frozen at − 80 degrees Celsius. This will be transported to Dr. Nichols’ lab.

##### Human papillomavirus testing

P16 testing (which is an excellent surrogate marker of HPV status) is required for enrollment. This will be done through the routine pathology laboratories as per current routine clinical care. The accompanying biomarker study will determine HPV status by real-time PCR, not for the purposes of randomization, but to confirm the accuracy of p16 results and also for subtyping of HPV strain. Pre-treatment FFPE primary site biopsy specimens will be retrieved in 10 slides 8 μm thick from the FFPE blocks. DNA will be extracted from the specimens for HPV testing by real-time PCR.

##### Whole genome sequencing analysis

DNA will be extracted either from fresh tumor or from formalin fixed specimens for patients undergoing TOS, as well as 10 mL of venous blood drawn prior to the initiation of treatment. Specimens yielding DNA of adequate quantity and quality (> 5 μg, OD between 1.8 and 2.0) will be subjected to high-throughput sequencing and gene copy number.

## Ethical considerations

The Principal Investigator will obtain ethical approval and clinical trial authorization by competent authorities according to local laws and regulations.

### Institutional Review Board (IRB) / Research Ethics Board (REB)

The protocol (and any amendments), the informed consent form, and any other written information to be given to subjects will be reviewed and approved by a properly constituted Institutional Review Board (IRB)/Research Ethics Board (REB), operating in accordance with the current federal regulations (e.g., Canadian Food and Drug Regulations (C.05.001); US Code of Federal Regulations (21CFR part 56)), ICH GCP and local regulatory requirements. A letter to the investigator documenting the date of the approval of the protocol and informed consent form will be obtained from the IRB/REB prior to initiating the study. Any institution opening this study will obtain REB IRB/REB approval prior to local initiation.

### Informed consent

The written informed consent form is to be provided to potential study subjects (Additional file 5) should be approved by the IRB/REB and adhere to ICH GCP and the ethical principles that have their origin in the Declaration of Helsinki. The investigator is responsible for obtaining written informed consent from each subject, or if the subject is unable to provide informed consent, the subject’s legally acceptable representative, prior to beginning any study procedures and treatment(s). The investigator should inform the subject, or the subject’s legally acceptable representative, of all aspects of the study, including the potential risks and benefits involved. The subject will be given ample time and opportunity to ask questions prior to deciding about participating in the study and be informed that participation in the study is voluntary and that they are completely free to refuse to enter the study or to withdraw from it at any time, for any reason.

The informed consent will be signed and dated by the subject, or the subject’s legally acceptable representative, and by the person who conducted the informed consent discussion. A copy of the signed and dated written informed consent form will be given to the subject or the subject’s legally acceptable representative. The process of obtaining informed consent will be documented in the patient source documents.

### Confidentiality

The names and personal information of study participants will be held in strict confidence. All study records (CRFs, safety reports, correspondence, etc.) will only identify the subject by initials and the assigned study identification number. The data coordinator will maintain a confidential subject identification list (Master List) during the course of the study. Access to confidential information (i.e., source documents and patient records) is only permitted for direct subject management and for those involved in monitoring the conduct of the study (i.e., Sponsors, CRO’s, representatives of the IRB/REB, and regulatory agencies). The subject’s name will not be used in any public report of the study.

### Data storage

All data will be stored on REDCap [25] a secure web application for building and managing online databases commonly used in the clinical trials research community. Ongoing auditing will be performed by the LRCP clinical trials unit, independent from the trial investigators and sponsor. All of the biomarker studies described in this section will be performed in Dr. Anthony Nichols’ laboratory located at the LRCP, in London, Ontario. All specimens will be labeled solely with the patient’s unique study identifier number, stored in a secure facility at LHSC, and will be accessed only by clinical trial staff.

### Adverse events

The severity of adverse events will be evaluated using the Common Terminology Criteria for Adverse Events (CTCAE) version 4.0 grading scale [26]. Any grade 4 or 5 adverse event that is definitely, probably, or possibly the result of protocol treatment must be reported to the Principal Investigator and Central Office within 24 h of discovery. The Serious Adverse Event (SAE) report form is to be completed with all available information and uploaded to the REDCap SAE page. The Central Office must be notified by email or telephone that a new SAE form has been uploaded into REDCap. It is the responsibility of each local Principal Investigator to report all SAEs to their REB as per local REB requirements. The Principal Investigator should also comply with the applicable regulatory requirement(s) related to the reporting of unexpected serious adverse drug reactions to the regulatory authority (ies).

## Data sharing statement

De-identified participant data from this trial will not be shared publicly, however, the full protocol will be published along with the primary analysis of the outcomes.

## Protocol ammendments and trial publication

Trial registration elements are summarized in Additional file 6. Any modifications to the trial protocol must be approved and enacted by the principal investigator (Current version: 1.3 on March 15, 2019). Protocol amendments will communicated to all participating centres, investigators, IRBs, and trial registries by the principal investigator. Any communication or publication of trial results will be led by the principal investigator, and is expected to occur within 1 year of the primary analysis. Trial results will remain embargoed until conference presentation of an abstract or until information release is authorized. Authorship of the trial abstract and ultimately the full manuscript will be decided by the principal investigator at the time of submission. Professional writers will not be used for either abstract or manuscript preparation.

## Discussion

Patients with HPV+ OPC have substantially better treatment response and OS than HPV- disease [3]. This has spurred increasing interest in de-escalation of therapy with the hopes of reducing treatment-related toxicity.

ORATOR2 is a multi-center phase II trial that aims to randomize 140 patients to two de-escalated interventions: de-escalated radiotherapy (± chemotherapy) or TOS (± adjuvant radiotherapy). The primary radiotherapy arm is based on the chemoradiation arm of HN002 [15] and the TOS arm is partially based on the treatment paradigm of ECOG-3311 [16].

This trial will assess the safety of both of these de-escalation approaches, comparing each to historical controls, and will allow for comparisons of OS, PFS and QOL between the two interventions. There is a paucity of data comparing primary RT and primary surgical approaches to oropharyngeal cancers. To date, the only randomized study examining this question is ORATOR, which enrolled patients regardless of HPV status, and included a small number of patients. ORATOR2 will allow for an assessment of both approaches in the early T-stage HPV+ patient population. Furthermore, the ORATOR trial can be used for additional historical controls, with a planned comparison between HPV+ patients in ORATOR (who were treated with more aggressive approaches) and ORATOR2 to assess differences in QOL and time-to-event outcomes.

A recruitment video has been created to help patients and the public understand the rationale and design behind the trial, and to help reach the target accrual (www.orator2.com).

Results of ORATOR2 are expected to provide data that will help guide treatment decisions in the management of HPV-associated OPC, which remains one of the most contentious issues in head and neck oncology.

## Supplementary information

**Additional file 1.** Treatment Planning Guidelines.

**Additional file 2.** Surgical Credentialling Form.

**Additional file 3.** Schedule of Enrolment, Interventions and Assessments.

**Additional file 4.** Lab Guidelines.

**Additional file 5.** Study Information and Informed Consent Form.

**Additional file 6.** World Health Organization Trial Registration Dataset.

## Data Availability

Not applicable.

## Abbreviations

AJCC: American Joint Committee on Cancer
CRF: Case report form
CRT: Chemoradiation
CT: Computed Tomography
CTC-AE: Common Toxicity Criteria for Adverse Events
ECOG: Eastern Cooperative Oncology Group
ENE: Extranodal extension
EORTC H&N35: European Organisation for Research and Treatment Quality of Life Questionaire, Head and neck module
EORTC QLQ-C30: European Organization for Research and Treatment of Cancer Quality of Life Questionnaire, Core Module
FDG: Fluorodeoxyglucose
HPV: Human papillomavirus
HPV-: Human papillomavirus negative
HPV+: Human papillomavirus positive
IMRT: Intensity modulated radiotherapy
LHSC: London Health Sciences Centre
LN: Lymph nodes
LRCP: London Regional Cancer Program
LVI: Lymphovascular invasion
MDADI: MD Anderson Dysphagia Inventory
NCI-CTC: National Cancer Institute Common Toxicity Criteria
NDII: Neck Dissection Impairment Index
OAR: Organ at risk
OPC: Oropharyngeal squamous cell carcinoma
OS: Overall survival
PCR: Polymerase Chain Reaction
PET: Positron-Emission Tomography
PFS: Progression-free survival
PNI: Perineural invasion
PNQ: Patient Neurotoxicity Questionnaire
QA: Quality Assurance
QOL: Quality of Life
RP: Retropharyngeal nodes
RT: Radiotherapy
TLM: Transoral laser microsurgery
TORS: Transoral robotic surgery
TOS: Transoral surgery
VHI-10: Voice Handicap Index-10
VMAT: Volumetric Modulated Arc Therapy
