# Supplementary material for: Treatment de-escalation for HPV-associated oropharyngeal squamous cell carcinoma with radiotherapy vs. trans-oral surgery (ORATOR2): study protocol for a randomized phase II trial
Source: BMC Cancer. 2020 Feb 14;20:125. doi: 10.1186/s12885-020-6607-z (PMC7023689; doi:10.1186/s12885-020-6607-z)
Supplement: Supplementary file 1 — Additional file 1. Treatment Planning Guidelines. [file 12885_2020_6607_MOESM1_ESM.docx]

# Additional file 1: TREATMENT PLANNING GUIDELINES

**Table A1. Dose constraints, planning priority and contouring definitions for organs at risk (OARs)**

| **OAR** | **Dose constraint** | **Planning Priority** | **Contouring definition** |
| --- | --- | --- | --- |
| **Spinal cord** | 48 Gy point dose  45 Gy to 0.1 cc | 1 | Cranial-cervical junction to T3/4 |
| **Spinal cord prv** | 52 Gy to 0.1 cc |  | Spinal cord + 5 mm in all directions. |
| **Brainstem** | 54 Gy point dose  50 Gy to 0.1 cc | 2 | From the top of the midbrain to the cranial-cervical junction. |
| **Brainstem prv** | 60 Gy to 0.1 cc |  | Brainstem + 5 mm in all directions |
| **Lips** | Mean < 20 Gy | 8 | Each should be contoured as a 2 cm structure in the cranio-caudal direction, and extend laterally to the commissures |
| **Oral cavity*** | Mean < 30 Gy | 9 | The anterior 2/3 of the tongue, floor of mouth, the buccal mucosa, and palate |
| **Parotid glands*** | Mean < 26 Gy | 4 - contralateral  11 - ipsilateral | Contoured bilaterally including the accessory lobes, not to overlap with the CTVs |
| **Mandible*** | Max < 66 Gy | 10 | Entire bony mandible |
| **Larynx*** | Max <45 Gy | 5 | A triangular volume extending from the inferior aspect of the hyoid to the superior aspect of the cricoid, anteriorly to include the anterior commissure and posteriorly to include the arytenoids. It does not include the suprahyoid epiglottis. |
| **Pharyngeal constricTOS*** | Mean <40 Gy for pharyngeal constrictor outside of the PTVs | 6 | Posterior pharyngeal wall, extending from the level of the inferior pterygoid plates to the cricoid. |
| **Submandibular gland*** | Mean < 39 Gy | 7 - contralateral  12 - ipsilateral | Both glands are to be contoured in their entirety, based on its appearance on CT. |

**Maximum doses will often be exceeded if the PTV overlaps with, or is in close proximity to, these structures. For example, if the contralateral level IB nodal group is within one of the PTVs, then the contralateral submandibular gland dose will be higher than the dose listed here.*

*Planning priority #3 is the PTVs (in descending order of dose, with highest dose level being the highest priority)*
