# Supplementary material for: Treatment de-escalation for HPV-associated oropharyngeal squamous cell carcinoma with radiotherapy vs. trans-oral surgery (ORATOR2): study protocol for a randomized phase II trial
Source: BMC Cancer. 2020 Feb 14;20:125. doi: 10.1186/s12885-020-6607-z (PMC7023689; doi:10.1186/s12885-020-6607-z)
Supplement: Supplementary file 2 — Additional file 2. Surgical Credentialling Form. [file 12885_2020_6607_MOESM2_ESM.docx]

# Additional file 2: Surgical Credentialling Form

Please check the item that best describes the scope of your practice:

___ General Otolaryngology

___ Head and Neck Surgery without fellowship training

___ Head and Neck Surgery with fellowship training (≥ 1 year)

Necessary criteria: Head and Neck Surgery with fellowship

2. Please estimate the number of neck dissections you perform per year. _______

Minimal criteria: 30 neck dissections / year

3. Please estimate the number of transoral endoscopic surgical procedures you perform each year

 _______

Minimal criteria: 20/year

4. Have you performed a minimum number of 20 cases of transoral excision for oropharyngeal carcinoma as the primary surgeon?

YES_______ NO_______

Minimal criteria: 20 cases

5. Have you performed at least 5 transoral resections of oropharyngeal carcinoma in the past 12 months?

YES_______ NO_______

Minimal criteria: 5 cases

6. Please contact study contact study coordinator Susan Archer to upload the operative notes and pathology reports for 10 transoral oropharyngeal cancer cases, including at least one tonsil and one tongue-base primary tumor.

7. If there are other surgeons at your institution who will be participating in this program, have they also completed one of these forms?

YES_______ NO_______
