# Supplementary material for: Treatment de-escalation for HPV-associated oropharyngeal squamous cell carcinoma with radiotherapy vs. trans-oral surgery (ORATOR2): study protocol for a randomized phase II trial
Source: BMC Cancer. 2020 Feb 14;20:125. doi: 10.1186/s12885-020-6607-z (PMC7023689; doi:10.1186/s12885-020-6607-z)
Supplement: Supplementary file 3 — Additional file 3. Schedule of Enrolment, Interventions and Assessments. [file 12885_2020_6607_MOESM3_ESM.docx]

# Additional file 3: Schedule of Enrolment, Interventions and Assessments

|  | **STUDY PERIOD** | | | | | | | | | | | | | | | | | | | | | | | | | |
| --- | --- | --- | --- | --- | --- | --- | --- | --- | --- | --- | --- | --- | --- | --- | --- | --- | --- | --- | --- | --- | --- | --- | --- | --- | --- | --- |
|  | Enrolment | Allocation | **Post-allocation** | | | | | | | | | | | | | | | | | | | | | | | |
|  |  |  | **Week from treatment start** | | | | | | | | | **Months post-treatment completion** | | | | | | | | | | | | | | |
| **TIMEPOINT** | -t1 | 0 | 1 | 2 | 3 | 4 | 5 | 6 | 7 | 8 | 9 | 3 | 4 | 6 | 9 | 12 | 15 | 18 | 21 | 24 | 30 | 36 | 42 | 48 | 54 | 60  (Close-out) |
| **ENROLMENT:** |  |  |  |  |  |  |  |  |  |  |  |  |  |  |  |  |  |  |  |  |  |  |  |  |  |  |
| Eligibility Screen | X |  |  |  |  |  |  |  |  |  |  |  |  |  |  |  |  |  |  |  |  |  |  |  |  |  |
| Informed consent | X |  |  |  |  |  |  |  |  |  |  |  |  |  |  |  |  |  |  |  |  |  |  |  |  |  |
| Allocation |  | X |  |  |  |  |  |  |  |  |  |  |  |  |  |  |  |  |  |  |  |  |  |  |  |  |
| **INTERVENTIONS:** |  |  |  |  |  |  |  |  |  |  |  |  |  |  |  |  |  |  |  |  |  |  |  |  |  |  |
| Primary RT |  |  |  |  |  |  |  |  |  |  |  |  |  |  |  |  |  |  |  |  |  |  |  |  |  |  |
| Primary TOS |  |  | X |  |  |  |  |  |  |  |  |  |  |  |  |  |  |  |  |  |  |  |  |  |  |  |
| **ASSESSMENTS:** |  |  |  |  |  |  |  |  |  |  |  |  |  |  |  |  |  |  |  |  |  |  |  |  |  |  |
| History and Physical Examination***^†^*** |  |  |  |  |  |  |  |  |  |  |  | X |  | X | X | X | X | X | X | X | X | X | X | X | X | X |
| Imaging***^‡^*** | X |  |  |  |  |  |  |  |  |  |  |  |  |  |  | X |  |  |  |  |  |  |  |  |  |  |
| CT neck or PET-CT to assess for residual nodes (Arm 1 only) |  |  |  |  |  |  |  |  |  |  |  |  | X |  |  |  |  |  |  |  |  |  |  |  |  |  |
| Dental Assessment | X |  |  |  |  |  |  |  |  |  |  |  |  |  |  |  |  |  |  |  |  |  |  |  |  |  |
| Audiogram, with CTCAE grade assessment | X |  |  |  |  |  |  |  |  |  |  |  |  |  |  | X |  |  |  |  |  |  |  |  |  |  |
| CTCAE Toxicity Assessment |  | X | Weekly during RT | | | | | | | | | X |  | X | X | X | X | X | X | X | X | X | X | X | X | X |
| Bloodwork | X |  | As per institutional standard of care for chemotherapy patients only | | | | | | | | |  |  |  |  | X |  |  |  |  |  |  |  |  |  |  |
| QOL Questionaires^ф^ (excluding PNQ) |  | X |  |  |  |  |  |  |  |  |  |  |  | X |  | X |  | X |  | X | X | X | X | X | X | X |
| PNQ |  | X |  |  |  |  |  |  |  |  |  |  |  |  |  | X |  |  |  |  |  |  |  |  |  |  |

**^†^** Physical examination includes laryngopharyngoscopy **^‡^** Imaging consists of CT of neck and chest **OR** MRI of neck and CT of the chest **OR** whole body PET-CT
