# Supplementary material for: Treatment de-escalation for HPV-associated oropharyngeal squamous cell carcinoma with radiotherapy vs. trans-oral surgery (ORATOR2): study protocol for a randomized phase II trial
Source: BMC Cancer. 2020 Feb 14;20:125. doi: 10.1186/s12885-020-6607-z (PMC7023689; doi:10.1186/s12885-020-6607-z)
Supplement: Supplementary file 4 — Additional file 4. Lab Guidelines. [file 12885_2020_6607_MOESM4_ESM.docx]

# additional file 4: LAB GUIDELINES

All blood and tumor samples will be labeled solely with each patient’s unique study number. No identifying data will be attached.

**For patients treated at London Health Sciences Centre:**

Blood samples: 10mL of blood shall be drawn pre operatively into a heparinized (green top) tube and directly transported to Dr. Nichols lab (address below).

Tumour samples: At the end of the study pre-treatment formalin fixed paraffin embedded (FFPE) primary site biopsy specimens will be sectioned, producing 10 slides 8um thick and three 1mm core punch biopsies of the tumor tissue within the FFPE blocks will be collected into a 1.5 ml eppendorf tube. These will be shipped to Dr. Nichols lab. For patients randomized to TOS with adequate available tumor, the main specimen will be taken to pathology frozen section room and a portion from the center of the specimen will be taken with the assistance of the pathologist and frozen at -80 degrees Celsius. This will be transported by Dr. Nichols research staff to his lab.

**For patients treated at all other study sites:**

Blood samples: 10mL of blood shall be drawn pre operatively into a heparinized (green top) tube. At the discretion of the local investigator the blood can be managed in two ways:

1. shipped immediately by courier on ice Dr. Nichols lab or:
2. DNA is immediately extracted (within 4 hours) using a Qiagen DNA extraction kit and stored in an eppendorf tube. DNA samples will be labeled with the ORATOR study number and can be stored at -4°C. Samples can either be shipped immediately to Dr. Nichols lab or in batches every 6 months.

Tumor Samples: At the end of the study pre-treatment formalin fixed paraffin embedded primary site biopsy specimens will be retrieved in 10 slides 8um thick as well as three 1mm core punch biopsies from the FFPE blocks. These will be shipped to Dr. Nichols lab.

**Dr. Nichols laboratory**

Attention Dr. John Barrett
Room A4-833, London Regional Cancer Program
790 Commissioners Rd. East
London, Ontario, Canada N6A 4L6
Phone: (519) 685-8600 ext. 53016 Fax: (519) 685-8616
