# Supplementary material for: Treatment de-escalation for HPV-associated oropharyngeal squamous cell carcinoma with radiotherapy vs. trans-oral surgery (ORATOR2): study protocol for a randomized phase II trial
Source: BMC Cancer. 2020 Feb 14;20:125. doi: 10.1186/s12885-020-6607-z (PMC7023689; doi:10.1186/s12885-020-6607-z)
Supplement: Supplementary file 5 — Additional file 5. Study Information and Informed Consent Form. [file 12885_2020_6607_MOESM5_ESM.docx]

# Additional file 5: Study Information and Informed Consent Form

**Radiation vs. Surgery for Treatment of Oropharyngeal Cancer**

A Phase II Randomized Trial of Treatment De-Escalation for HPV-Associated Oropharyngeal Squamous Cell Carcinoma: Radiotherapy vs. Trans-Oral Surgery (ORATOR II)

Study ID: ORATOR II

Study Doctor: Dr. ________________

Sponsor: London Regional Cancer Program

*If an REB approved French consent is not used at your institution remove this statement.*

Le formulaire de consentement est disponible en français sur demande.

**Emergency Contact Number** (24 hours / 7 days a week): _________________________

Non-Emergency contact numbers are at the end of this document under Contacts.

**Introduction**

You are being invited to participate in a clinical trial (a type of study that involves research). Clinical trials only include participants who choose to take part. You are invited to participate in this trial because you have cancer of the back of the tongue, the tonsils, or the upper throat. This area is called the oropharynx. Your tumor has been tested and found to have the presence of the human papillomavirus (HPV) within it. Patients with HPV related tumors have a high chance of cure, thus there is great interest in using less intensive therapy so that patients suffer less side effects.

This consent form provides you with information to help you make an informed choice. Please read this document carefully and take your time in making your decision. You may find it helpful to discuss it with your friends and family.

Taking part in this study is voluntary. You may choose not to take part or if you choose to participate may leave the study at any time without giving a reason. Deciding not to take part or deciding to leave the study later will not result in any penalty or any loss of benefits to which you are entitled.

**Background**

The usual treatment for your disease is radiation therapy, which is a beam of x-rays directed at the tumor and some of the lymph nodes in the neck. Radiation is delivered every day for 7 weeks, excluding weekends and holidays. Some patients require chemotherapy with the radiation, and some patients may even require surgery if the tumor or lymph nodes have not responded after radiation. However, as HPV related tumors are highly curable, there is great interest in using less intensive radiation and chemotherapy or minimally invasive surgery to decrease treatment side effects while maintaining high cure rates.

Health Canada, the regulatory body that oversees the use of drugs in Canada, has approved the sale and use of the chemotherapy agents used in this study to treat oropharyngeal cancer.

The research ethics board, which oversees the ethical conduct of research involving humans, has reviewed and accepted this study.

**Purpose**

The purpose of this study is to compare decreased doses of radiation therapy (RT) (with added chemotherapy if your doctor determines it’s necessary) with a surgical treatment called transoral surgery (TOS) to find out what effects the transoral surgery has on you and your oropharyngeal cancer. You will be offered a surgery through the mouth using either a surgical robot (called transoral surgery (TOS)), a laser (transoral laser microsurgery (TLM)) or a tool that is heated electrically to burn tissue (called electrocautery). TOS and TLM are new surgical approaches using either a robot or a laser to assist your surgeon in removing the tumor, potentially with fewer side effects than older surgical techniques. These approaches allow the surgeon to remove the tumor much more easily than with older techniques, without having to cut through the neck to access the primary tumor. Your doctor will discuss which surgery will be offered to you before you agree to participate in the study.

As part of the surgery, your surgeon will also need to remove some of the lymph nodes in your neck and may insert a temporary tube in your airway through your neck (called a tracheostomy).Some participants require radiation therapy and/or chemotherapy after surgery, depending on how advanced the tumor is found to be.

**Alternative Treatments**

You do not have to take part in this study in order to receive treatment. Other options may include, but are not limited to:

- Standard full dose radiation with or without chemotherapy
- Best supportive care*.* This type of care helps reduce pain, tiredness, appetite problems and other problems caused by the cancer. It does not treat the cancer directly, but instead tries to improve how you feel. Best supportive care tries to keep you as active and comfortable as possible.
- Other experimental studies may be available if you do not take part in this study.

Please talk to your study doctor about the known benefits and risks of these other treatment options before you decide to take part in this study. Your study doctor can also discuss with you what will happen if you decide not to undertake any treatment at this time.

You may get treatment at this centre and other centres even if you do not take part in the study.

**Expected Number of Participants**

Up to 140 people will take part in this study in Canada and Australia.

This study should take **10** years to complete and the results should be known in about 12 years.

Your study doctor will be informed of the results of this study once they are known.

**Assignment to a Group**

If you decide to participate you will be "randomized" into one of the study groups described below. Randomization means that you are put into a group by chance. It is like flipping a coin. There is no way to predict which group you will be assigned to. Neither you nor your doctor can choose what group you will be in. You will have a 50/50 chance of being placed in either group. You will be told which treatment you are to get.

**Group 1 (Experimental Treatment):** Low Dose Radiation Therapy ± Low Dose Chemotherapy

If you are randomized to Group 1 you will receive radiation, 5 days a week, Monday through Friday, for a total of 30 treatments. In some cases, the 30 treatments are delivered over 6 weeks. Each treatment will take approximately 20 minutes.

Some participants may require chemotherapy treatment as well, depending on how advanced their tumor is found to be. Chemotherapy is a type of drug called cisplatin,, delivered weekly for 6 cycles (1 cycle is 1 week). Chemotherapy is administered intravenously (by a needle in one of your veins). Occasionally other types of chemotherapy are used.

You will be evaluated 8-12 weeks after the completion of radiation therapy. If the study doctor detects that there is still a tumor or enlarged lymph nodes after radiation therapy you may also require surgery. This surgery (called salvage surgery) is standard of care for patients with cancer that does not completely go away with radiation and chemotherapy alone.

**Group 2 (Experimental Treatment):** Transoral Surgery (TOS) and Neck Dissection ± Low Dose Radiation Therapy

If you are randomized to Group 2 you will receive two procedures. The first procedure will remove the cancer in your throat (TOS or TLM). At the same time or within 2 weeks prior to this surgery, you will undergo a procedure to remove the lymph nodes in your neck, called a neck dissection. During the surgery, your surgeon may insert a temporary tube in your airway through your neck (called a tracheostomy). The surgical procedures will take about 3 hours.

Depending on the surgical findings, you may require treatment with radiation therapy after your surgery. This will be given in up to 30 treatments and delivered over 6 weeks. Each treatment will take approximately 20 minutes.

**Study Procedures**

**Non-Experimental Procedures**

The following tests will be done as part of this study. Some of these tests may be done as part of your standard care, in which case the results may be used. Some of these tests may be done more frequently than if you were not taking part in this study and some may be done solely for the purpose of the study. If the results show that you are not able to continue participating, your study doctor will let you know:

1. A radiotherapy planning CT scan for the creation of a mask placed over your head to keep your head in place during each radiation treatment (Group 1 participants).
   - physical examination and medical history
   - routine blood tests
   - pregnancy test for women who are able to become pregnant
   - Imaging: depending on your institutional standards, your routine imaging will include one or more or the following scans:
     - - Magnetic resonance imaging (MRI) of neck - an imaging technique that uses a strong magnet to produce pictures of areas inside the body. MRI is useful for assessing organs and other soft tissue, such as the inside of bones
       - Computed tomography (CT) scan of the neck and chest – a series of x-rays of the body from many angles that are turned into 3-dimensional pictures on a screen. CT scans often involve injecting a dye into your vein.
       - PET/CT Scan: A scan that performs a CT scan at the same time as a Positron Emission Tomography (PET) – a scan to help show how organs and tissues are working by tracing where a small amount of glucose (a sugar) that includes a tiny, harmless amount of radioactivity, goes in your body after it has been injected into one of your veins.
   - Dental evaluations
   - Audiogram (hearing test) before initiation of treatment

**Questionnaires**

You will be asked to fill out questionnaires before starting the study, and after your treatment at months 6, 12, 18, 24 then every 6 months thereafter for 5 years*,* to understand how your treatment and illness affect your quality of life. These questionnaires ask about how you are feeling and take about 10-20 minutes to complete. The information you provide is for research purposes only and will remain strictly confidential.

Some of the questions are personal; you may choose not to answer these if you wish. Even though you may have provided information on a questionnaire, these responses will not be reviewed by your health care team or study team. If you wish them to know this information, please bring it to their attention.

**Central Radiotherapy Review**

Copies of your radiation scans will be collected as part of this study. This is required for quality assurance and data management. The copies will be sent to the Quantitative Imaging for Personalized Cancer Medicine Platform in Toronto, Canada and kept for 1 year after the end of the study monitoring period when they will be destroyed.

To protect your identity, the information that will be on your radiation scans will be limited to your study number, which may include your initials.

**Mandatory Sample Collection**

The researchers doing this study need to do tests on samples (described below) to learn more information about oropharyngeal cancer.

The collection of these samples is a necessary part of this study and will be used only for these purposes. The samples will not be sold. Once these tests have been completed, any leftover samples will be destroyed. If you participate in this study it is possible that not enough tumor tissue will be left for other testing that may need to be done in the future. Please speak to your study doctor to discuss this possibility.

Certain types of genetic testing could have implications for you or your biological relatives. The researchers believe the chance these things will happen is very small, but cannot promise that they will not occur. Please ask your study doctor whether this might apply to you as a result of your participation in this study.

Hereditary genomic testing (to find out if cancer runs in your family) will not be done on these samples.

Reports about any research tests done with your samples will not be given to you or your study doctor. These reports will not be put in your medical records.

If you are a First Nations or an indigenous person who has contact with spiritual Elders, you may want to talk to them before you make a decision about this research study. Elders may have concerns about some research procedures including genetic testing.

**Tissue Collection (Required)**

You have already had a sample of your tumor taken to make the diagnosis of cancer. A portion of this sample will be sent to the researchers doing this study in London, Ontario. No further surgeries or biopsies are required of you for this purpose. If your biopsy or surgery were completed at another institution, signing this consent form means that you are consenting to the collection of your tissue sample, together with any related personal health information, from that institution. This sample will be used to do detailed testing for the presence of Human Papilloma Virus (HPV).

If you enter the study and receive the TOS surgery (Group 2), a further small sample will be taken from the tumor at the time the tumor is removed. This will be used for detailed testing for the presence of HPV and analyzing the genetic makeup of the tumor.

All mandatory samples will be sent to Dr. Anthony Nichols’ laboratory at the London Regional Cancer Program in London, Ontario, Canada.

**Blood Collection (Required)**

As part of the study, a blood sample will be taken by inserting a needle into a vein in your arm. Study staff will make every effort to take this sample at the same time as your routine blood work. 10 mL (approximately 1 teaspoon) of blood will be collected before study entry and this sample will be sent to a laboratory at the London Regional Cancer Program. Researchers will look at the DNA in the sample to see what features are unique to your tumor.

**Identification of Samples**

To protect your identity, the information that will be on your samples will be limited to your patient study number and date of collection.

**Withdrawal of Required Samples**

If you no longer want your samples to be used in this research, you should tell your study doctor. Your study doctor will notify the sponsor, who will ensure the samples are destroyed. If tests have already been done on your sample(s) it will not be possible to withdraw those results. However, no further testing will be done.

You may continue to participate in this main part of the study if you withdraw these required samples.

**Summary of Treatments, Tests and Procedures**

**Pretreatment Evaluation for both Group 1 and 2 Participants**

| **Day** | **Tests, Procedures and Treatments** |
| --- | --- |
| Within 12 weeks prior to randomization | - CT neck and chest or MR neck and CT chest or full body PET/CT |
| Within 8 weeks prior to randomization | - History and physical examination - HPV Tumor Status |
| Within 4 weeks prior to randomization | - Routine blood tests - Dental Evaluation - Pregnancy Test |
| Before Initiation of Treatment | - Audiogram - Study blood for DNA testing - Complete questionnaires |

**GROUP 1 STUDY PARTICIPANTS**

**Study Treatment**

| **Day** | **Tests, Procedures and Treatments** |
| --- | --- |
| 5 days a week (Monday to Friday) for 6 weeks | - Radiation Therapy treatment, potentially with chemotherapy |

**During Treatment Evaluations**

| **Day** | **Tests, Procedures and Treatments** |
| --- | --- |
| Weekly during Radiation Therapy (RT) | - Physical exam and monitor any side effects you may be experiencing as a result of the treatment. |
| 8-12 Weeks after Completion of RT | - CT Scan of Neck and/or a PET/CT Scan of the neck |
| Every 3 months for first 2 years then every 6 months until 5 years | - History and physical examination including laryngopharyngoscopy (looking at the back of the throat using a small camera inserted in the nose) - Assessment of side effects you may be experiencing |
| Every 6 months for 5 years | - Completion of Quality of Life Questionnaires |
| At 12 months from first day of RT | - Completion of Quality of Life Questionnaires - CT Scan of neck and chest or PET/CT scan - Audiogram - Bloodwork |

**GROUP 2 STUDY PARTICIPANTS**

**Study Treatment**

| **Day** | **Tests, Procedures and Treatments** |
| --- | --- |
|  | - Transoral surgery (TOS) to remove tumor from throat |
| Same day or within 2 weeks prior to TOS | - Removal of lymph nodes in neck |
| Within 6 weeks of surgery | - Depending on the surgical findings, you may require treatment with radiation therapy with or without chemotherapy after your surgery |

**During Treatment Evaluations**

| **Day** | **Tests, Procedures and Treatments** |
| --- | --- |
| 2 weeks following surgery | - Routine post-operative assessment |
| Every 3 months for first 2 years then every 6 months until 5 years | - History and physical examination including laryngopharyngoscopy (looking at the back of the throat using a small camera inserted in the nose) - Assessment of side effects you may be experiencing |
| Every 6 months for 5 years | - Completion of Quality of Life Questionnaires |
| At 12 months after surgery | - Completion of Quality of Life Questionnaires - CT Scan of neck and chest or PET/CT scan - Audiogram |

**Responsibilities**

If you choose to participate in this study, you will be expected to:

- Tell your study doctor about your current medical conditions;
- Tell your study doctor about all prescription and non-prescription medications and supplements, including vitamins and herbals, and check with your study doctor before starting, stopping or changing any of these. This is for your safety as these may interact with the treatment you receive on this study;
- Tell your study doctor if you are thinking about participating on another research study;
- Tell your study doctor if you become pregnant or father a child while participating on this study

**Length of Participation**

If you are randomized to group 1, your treatment with standard radiation or chemo-radiation will last approximately 6 weeks, and you may require surgery afterward if any disease remains. If you are randomized to group 2, you will have the two surgical procedures, and you may require radiation therapy (and maybe chemotherapy) over 6 weeks.

After your last study treatment, you will be asked to come back to the hospital every 3 months for 2 years and then every 6 months from years 3-5. You may be seen more often if your study doctor determines that this is necessary, or if your cancer comes back.

No matter which group you are randomized to, and even if you stop treatment early, we would like to keep track of your health for the next 5 years to look at the long-term effects of the study treatments. This would be done by contacting you for a follow up visit or by contacting you by phone every 3 months for 2 years and then every 6 months from years 3-5.

**Early End to Participation**

Your participation in the trial may be stopped early, for reasons such as:

- The treatment does not work for you and your cancer comes back or gets worse.
- You are unable to tolerate the study treatment
- You no longer wish to participate.
- New information shows that the study treatment is no longer in your best interest.
- Your study doctor no longer feels this is the best treatment for you.
- The sponsor decides to stop the study
- If you become pregnant

If your participation in the study is stopped your study doctor will provide information about how to stop safely.

**Risks of Participation**

Participating in this study will put you at risk for the side effects listed below. You should discuss these with your study doctor. As with any treatment additional unexpected and sometimes serious side effects are a possibility.

Your study doctor will watch you closely to see if you have side effects. When possible, drugs will be given to you to make side effects less serious and more tolerable. Many side effects go away shortly after your treatment is stopped but in some cases side effects can be serious, long-lasting, permanent, or may even cause death.

If you experience serious side effects that require treatment between regular clinic/hospital visits, it is important that you make every effort to return to the clinic/hospital where your treatment was given. If you need immediate treatment and are unable to return to the clinic/hospital where you received your treatment, you should go to the nearest Emergency department and your study doctor should be contacted as soon as possible.

The risks and side-effects of the standard, or usual treatment will be explained to you as part of your standard care and are therefore not listed.

There is a chance that a decreased dose of radiation will not work as well as the full dose of radiation.

**Risks and side effects related to the TOS and Neck Dissection include:**

**Very likely (*21% or more, or higher than a 1 in 5 risk)*:**

- Throat pain making it difficult to swallow
- Weight loss
- Temporary nasal voice quality
- Neck numbness
- Temporary tongue swelling

**Less likely (*5 to 20% or between a 1 in 5 and 1 in 20 risk*):**

- Bruising or burns to the lips or gums
- Tooth Injury
- Weight loss
- Permanent nasal voice quality
- Weakness of your lower lip
- Weakness of your shoulder or tongue
- Difficulty breathing
- Difficulty swallowing. In some cases, patients might need a long-term feeding tube, and can be at risk of inhaling food or liquids into the lungs.
- Bleeding from the throat or neck
- Stroke

**Rarely (1 – 4%)**:

- Dehydration
- Aspiration pneumonia (infection in the lungs because of saliva getting into the lungs)
- Injury to the breathing passages
- long-term requirement for a feeding tube
- Injury to the nerves of the tongue and neck
- Death due to a severe complication of surgery

Our estimates of the risks associated with TOS are based on small studies. The actual risks may prove to be higher when more information about TOS becomes available in the future. Specifically, the risk of fatal bleeding caused by surgery, or other serious side effects caused by surgery, might prove to be higher as larger studies of TOS become available.

**Reproductive Risks**

The treatments used in this study may harm an unborn baby (fetus). You must not become pregnant or father a baby during study treatment and for 6 months after your last treatment.

Your study doctor will discuss methods with you to ensure that you do not become pregnant or father a baby during the study.

Women should not nurse (breastfeed) a baby while taking study treatment and for 6 months after the last treatment because the treatments used in this study may be present in breast milk and could be harmful to a baby.

If you become pregnant or father a child during this study, or for 6 months after you stop treatment, then you should immediately notify your study doctor. Your study doctor will let the sponsor know about the pregnancy.

**Data Safety Monitoring Committee**

A Data Safety Monitoring Board/Committee, an independent group of experts, will be reviewing the data from this research throughout the study.

**Benefits**

If you agree to take part in this study, there may or may not be direct benefit to you. The researchers hope the information learned from this study will help other patients in the future.

**Confidentiality**

Records identifying you at this centre will be kept confidential and, to the extent permitted by the applicable laws, will not be disclosed or made publicly available, except as described in this consent document.

Studies involving humans now routinely collect information on race and ethnicity as well as other characteristics of individuals because these characteristics may influence how people respond to different medications. Providing information on your race or ethnic origin is voluntary.

Authorized representatives of the following organizations may look at your original (identifiable) medical/clinical study records at the site where these records are held, for quality assurance (to check that the information collected for the study is correct and follows proper laws and guidelines):

- London Regional Cancer Program, the sponsor of the trial;
- The Ontario Cancer Research Ethics Board, which oversees the ethical conduct of this study in your clinic/hospital;

Authorized representatives of the above organizations and the organization listed below may receive information related to the study from your medical/clinical study records for quality assurance and data analysis. Your name or other information that may identify you will not be used. The records received by these organizations may contain your study participant code, partial initials, month and year of your birth and your sex.

- Dr. Anthony Nichols’ Laboratory, London Regional Cancer Program
- Quantitative Imaging for Personalized Cancer Medicine Platform (QIPCMP) in Toronto, Canada

All of the organizations listed in the above confidentiality sections are required to have strict policies and procedures to keep the information they see or receive about you confidential, except where disclosure may be required by law. The study doctor will ensure that any personal health information collected for this study is kept in a secure and confidential location as required by law. There are federal and provincial laws that these organizations must comply with to protect your privacy

If the results of this study are published, your identity will remain confidential. It is expected that the information collected during this study will be used in analyses and will be published/ presented to the scientific community at meetings and in journals.

Even though the likelihood that someone may identify you from the study data is very small, it can never be completely eliminated.

A copy of this signed and dated consent form may be included in your health record/hospital chart.

Your family doctor/health care provider will be informed that you are taking part in a study so that you can be provided with appropriate medical care. If you do not want your family doctor/health care provider to be informed, please discuss with your study doctor.

A wallet card will be provided to you with information about how to contact the study staff when required.

Your de-identified data from this study may be used for other research studies. If your study data is shared with other researchers, information that links your study data directly to you will not be shared.

**Registration of Clinical Trials**

A description of this clinical trial will be available on [www.clinicaltrials.gov](http://www.clinicaltrials.gov). This website will not include information that can identify you. You can search this website at any time.

**Costs**

The costs of your medical treatment will be paid for by your provincial medical plan to the extent that such coverage is available. There may be extra costs that are not covered by your medical plan that you will have to pay yourself; some examples may be physiotherapy or certain pain medications.

Taking part in this study may result in added costs to you (i.e. transportation, parking, meals, or unpaid leave from work). You may have to pay for medication prescribed to treat or prevent side effects, and you may have to visit the hospital more often than if you were not participating in this study.

**Compensation**

You will not be paid for taking part in this study.

*You will be reimbursed for study-related expenses such as specify, e.g., parking, etc.*

*Note: this statement may be removed/revised as per centre requirements.*

It is possible that the research conducted using your samples and/or study data may eventually lead to the development of new diagnostic tests, new drugs or other commercial products. There are no plans to provide payment to you if this happens.

In the case of research-related side effects or injury, medical care will be provided by your doctor or you will be referred for appropriate medical care.

**Rights**

You will be told, in a timely manner, about new information that may be relevant to your willingness to stay in this study.

If you decide to stop participating in the study or if your participation has been stopped, your doctor will discuss other options with you and continue to treat you with the best means available.

You may withdraw your permission to use your personal health information for this study at any time by letting the study doctor know. However, this would also mean that you withdraw from the study. Your study data that was recorded before you withdrew will be used but no information will be collected or sent to the sponsor after you withdraw your permission.

Your rights to privacy are legally protected by federal and provincial laws that require safeguards to ensure that your privacy is respected.

By signing this form you do not give up any of your legal rights against the investigators, sponsor or involved institutions for compensation, nor does this form relieve the investigators, sponsor or involved institutions of their legal and professional responsibilities.

You will be given a copy of this signed and dated consent form prior to participating in this study.

**Conflict Of Interest**

The doctor treating you also may be the doctor in charge of the study.

This centre is receiving funds from the London Regional Cancer Program, the sponsor of the study, to help offset the costs of conducting this research. The researchers at this centre will not receive any direct benefit for conducting this study.

If you would like additional information about the funding for this study, or about the role of the doctor in charge of this study, please speak to the study staff or to the Office of the Chair of the Ontario Cancer Research Ethics Board. (Contact information below)

**Contacts**

If you have questions about taking part in this study, or if you suffer a research-related injury, you should talk to your study doctor. Or, you can meet with the doctor who is in charge of the study at this institution. That person is:

| *Name* |  | *Telephone #* |
| --- | --- | --- |

If you have questions about your rights as a participant or about ethical issues related to this study, you can talk to someone who is not involved in the study at all. Please contact the Office of the Chair of the Ontario Cancer Research Ethics Board at:

| Telephone: 416-673-6648 |  | Toll Free: 1-866-678-6427 ext. 6648 |
| --- | --- | --- |

**Signatures**

- All of my questions have been answered,
- I understand the information within this informed consent form,
- I allow access to my medical records and specimens as explained in this consent form,
- I am aware of the risks to me of participating in the study and the risks to the fetus if I become pregnant or father a child during this study,
- I do not give up any of my legal rights by signing this consent form,
- I agree to take part in this study.

| Signature of Participant |  | Printed Name |  | Date |
| --- | --- | --- | --- | --- |

| Signature of Person Conducting the Consent Discussion |  | Printed Name |  | Date |
| --- | --- | --- | --- | --- |

**Participant Assistance**

**Complete the following declaration only if the participant is unable to read:**

- The informed consent form was accurately explained to, and apparently understood by, the participant, and
- Informed consent was freely given by the participant

| Signature of Impartial Witness |  | Printed Name |  | Date |
| --- | --- | --- | --- | --- |

**Complete the following declaration only if the participant has limited proficiency in the language in which the consent form is written and interpretation was provided as follows:**

- The informed consent discussion was interpreted by an interpreter, and
- A sight translation of this document was provided by the interpreter as directed by the research staff conducting the consent.

**Interpreter Declaration and Signature:**

By signing the consent form I attest that I provided a faithful interpretation for any discussion that took place in my presence, and provided a sight translation of this document as directed by the research staff conducting the consent.

| Signature of Interpreter |  | Printed Name |  | Date |
| --- | --- | --- | --- | --- |
