# Supplementary material for: Treatment de-escalation for HPV-associated oropharyngeal squamous cell carcinoma with radiotherapy vs. trans-oral surgery (ORATOR2): study protocol for a randomized phase II trial
Source: BMC Cancer. 2020 Feb 14;20:125. doi: 10.1186/s12885-020-6607-z (PMC7023689; doi:10.1186/s12885-020-6607-z)
Supplement: Supplementary file 6 — Additional file 6. World Health Organization Trial Registration Dataset. [file 12885_2020_6607_MOESM6_ESM.docx]

# Additional file 6: World Health Organization Trial Registration Dataset

| **Item** | **Description** |
| --- | --- |
| Primary registry and trial identifying number | ClinicalTrials.gov : NCT03210103 |
| Date of registration in primary registry | July 6, 2017 |
| Secondary identifying numbers | NA |
| Source(s) of monetary or material support | London Health Sciences Foundation (philanthropic donations) and Ontario Institute of Cancer Research  Department of Otolaryngology – Head and Neck Surgery, University of Western Ontario |
| Primary sponsor | Lawson Health Research Institute |
| Secondary sponsor(s) | NA |
| Contact for public queries | Dr. David A. Palma |
| Contact for scientific queries | Dr. David A. Palma |
| Public title | Primary Radiotherapy Versus Primary Surgery for HPV-Associated Oropharyngeal Cancer (ORATOR2) |
| Scientific title | A Randomized Trial of Treatment De-Escalation for HPV-Associated Oropharyngeal Squamous Cell Carcinoma: Radiotherapy vs. Trans-Oral Surgery (ORATOR2) |
| Countries of recruitment | Canada |
| Health condition(s) or problem(s) studied | HPV-Associated Oropharyngeal Squamous Cell Carcinoma |
| Intervention(s) | Radiotherapy Arm: Radiation, with or without concurrent chemotherapy |
|  | TOS Arm: Transoral surgery and neck dissection, with or without adjuvant radiotherapy |
| Key inclusion and exclusion criteria | - Inclusion: Age 18 years or older; willing to provide informed consent; ECOG performance status 0-2; Histologically confirmed squamous cell carcinoma; P16 positive or HPV positive; Primary tumor site in the oropharynx (includes tonsil, soft palate, base of tongue, walls of oropharynx); Eligible for curative intent treatment, with likely negative resection margins at surgery (for patients where adequate transoral access is in question, they will first undergo an examination under anesthesia prior to randomization to ensure adequate exposure can be obtained); Smokers and non-smokers are included (Patients will be stratified by ,<10 pack years smoking history versus > or equal to 10 pack years); Tumor stage (AJCC 8th edition): T1 or T2; Nodal stage (AJCC 8th edition): N0, N1, or N2; For patients who may require chemotherapy (ie, patients with multiple lymph nodes positive or a single node more than 3 cm in size, in any plane) CBC/differential within 4 weeks prior to randomization with adequate bone marrow function, hepatic, and renal function defined as: Hemoglobin ≥ 80 g/L; Absolute neutrophil count ≥ 1.5 x 10 9/L, platelets ≥ 100 x 10 9/L, bilirubin ≤ 35 umol/L, AST or ALT ≤ 3 x the upper limit of normal; serum creatinine ≤ 130 umol/L or creatinine clearance ≥ 50 ml/min; Patients assessed at head and neck multidisciplinary clinic (with assessment by radiation oncologist and surgeon) and presented at multidisciplinary tumor board prior to randomization. |
|  | - Exclusion: Unambiguous clinical or radiological evidence of extranodal extension on pre-treatment imaging. This includes the presence of matted notes, defined as 3 or more nodes that are abutting with loss of intervening fat planes; Serious medical comorbidities or other contraindications to radiotherapy, chemotherapy or surgery; Prior history of head and neck cancer within 5 years; Prior head and neck radiation at any time; Metastatic disease; Inability to attend full course of radiotherapy or follow up visits; Prior invasive malignant disease unless disease-free for at least 5 years or more, with the exception of non-melanoma skin cancer; Pregnant or lactating women. |
| Study type | Randomized by permuted blocks sequence |
|  | No masking/blinding (open label) |
|  | Parallel assignment |
|  |  |
| Date of first enrolment | Feb 13, 2018 |
| Target sample size | 140 |
| Recruitment status | Recruiting |
| Primary outcome(s) | Overall survival |
| Key secondary outcomes | Progression-free survival; Quality of Life; Toxicity profile; Feeding tube rate; |
